# Long-Range Wireless Mesh Network for Weather Monitoring in Unfriendly Geographic Conditions

**DOI:** 10.3390/s110707141

**Published:** 2011-07-12

**Authors:** Manuel Toledano-Ayala, Gilberto Herrera-Ruiz, Genaro M. Soto-Zarazúa, Edgar A. Rivas-Araiza, Rey D. Bazán Trujillo, Rafael E. Porrás-Trejo

**Affiliations:** Department of Research and Postgrade Studies, Autonomus University of Querétaro, Cerro de las Campanas S/N, C.P. 76010 Querétaro, México; E-Mails: gherrera@uaq.mx (G.H.-R.); genaro.soto@uaq.mx (G.M.S.-Z.); erivas@uaq.mx (E.A.R.-A.); bazz.link@gmail.com (R.D.B.T.); porras@uaq.mx (R.E.P.-T.)

**Keywords:** wireless sensor networks, remote monitoring, embedded systems

## Abstract

In this paper a long-range wireless mesh network system is presented. It consists of three main parts: Remote Terminal Units (RTUs), Base Terminal Units (BTUs) and a Central Server (CS). The RTUs share a wireless network transmitting in the industrial, scientific and medical applications ISM band, which reaches up to 64 Km in a single point-to-point communication. A BTU controls the traffic within the network and has as its main task interconnecting it to a Ku-band satellite link using an embedded microcontroller-based gateway. Collected data is stored in a CS and presented to the final user in a numerical and a graphical form in a web portal.

## 1. Introduction

Wireless technologies have seen rapid development in recent years. One of the most popular applications of wireless technologies is for interpersonal communications, such as cellular phones, but in environmental monitoring and automation applications there also exists a greater potential for the use of these technologies. In some specific areas there exist target environments that are often too dangerous or inaccessible to humans or large robots and there are many challenges for deploying and maintaining wireless sensor networks (WSNs) in those unfriendly environments [1]. These facts, together with the advances in miniaturization of electronic devices have led to the emergence of specialized WSNs.

WSNs offer improved robustness and enhanced flexibility compared with wired systems and also allow the development of applications that could not be considered possible before, such as monitoring dangerous, hazardous, unwired, or difficult to access areas and locations. An example of an efficient WSN is presented in [2] where a water environment monitoring system based on a WSN for water surveillance in an artificial lake was developed. The system is formed by several monitoring nodes linked through the ZigBee protocol, where the data from each node is gathered by a base station that sends measurements such as pH and temperature of the water to a remote monitoring center via a GPRS network.

Over the last decade, WSNs have generated considerable enthusiasm within the networking researcher community in order to apply them to a wide range of applications, such as environmental monitoring, military target tracking, weather forecasting, home automation, intrusion detection, *etc.* [3–11]. However there are several key issues to address when selecting a suitable technology for wireless data transfer, which are determined by the application target. These include communication reliability, transmission range, data transfer rates and power consumption. WSNs are often classified in terms of transmission range, of which the standards for wireless PAN (Personal Area Network), infrared data association (IrDA), IEEE 802.15.1 (Bluetooth), and IEEE 802.15.4 (ZigBee) are the most widely used short-range technologies. Mid-range wireless local area networks (LAN) comprise IEEE 802.11b (WiFi) which is able to handle very high data rates at the expense of a higher power demand, while long-distance wireless networks include satellite communication and cellular phone systems, such as GSM/GPRS and CDMA.

Long-range monitoring and automation applications demand new and innovative strategies and wireless network topologies in order to guarantee a minimal quality of service (QoS) while minimizing power consumption and operation costs. Long distance communication in general is more expensive and is subject to interferences; moreover in unfriendly access zones, power delivery is not available, this leads to the need for efficient use of the resources. This represents a challenge for WSNs [12] and specially collecting raw data for environmental monitoring applications can be difficult when this collecting task involves elements which are either widespread, their positions are subject to change [13] or there is a high-energy consumption involved [9]. In this way, the trend points to minimize power consumption in WSNs, permiting the system to operate with batteries and in many applications, the power is supplied with the help of the energy harvested from the environment where the device is located.

In this paper, some challenges mentioned above for a long-range wireless mesh network for weather monitoring in unfriendly geographic conditions are contemplated. In addition, a hybrid wireless network involving the use of a free ISM band interconnected to a KU satellite link by an embedded microcontroller-based gateway is presented.

## 2. General Overview of the System

**Figure 1.**
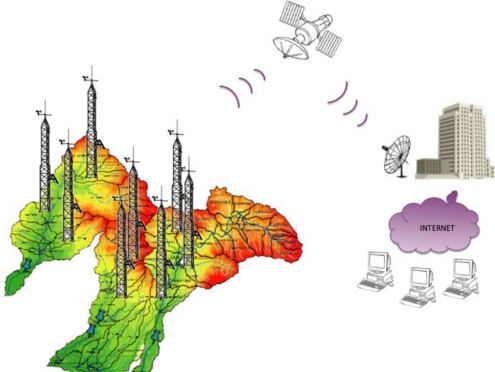
General overview of the proposed system, on the left side the ISM network, on the center and right side the KU Band Satellite link and on the right side the monitoring center.

The proposed system is composed of three main parts: Remote Terminal Units (RTU), Base Terminal Units (BTU), and the Central Server (CS). The RTUs are designed based on a high performance microcontroller and designed to monitor the climatic variables: Air Temperature (AT), Relative Humidity (RH), Barometric Pressure (BP), Solar Radiation (SR), Wind Direction (WD), Wind Speed (WS) and Rain (R). Each RTU interacts with other RTUs as they integrate a wireless local area network (WLAN); they are responsible for collecting data from the meteorological sensors and then for sending this data to the BTUs by Radio Frequency (RF) in the 900 MHz frequency band designed for Industrial Scientific and Medical (ISM) applications. The BTU has the task to address the requests from the RMS to the corresponding RTU and to send back the data collected by them. This information is sent with intervals of 10 min through a satellite link in the Ku band. Data sent by the BTUs is stored in the CS where historical data is generated and presented to the final user in a portal web in Graphic User Interface (GUI). Figure 1 illustrates a general overview of the system.

## 3. Remote Terminal Unit (RTU)

A RTU is based on the 16-bit high performance microcontroller (MCU) PIC18F8723 which has among other features up to 16 channels of bit resolution analog to digital converter (ADC), two enhanced addressable USART modules and two master synchronous serial port (MSSP) modules supporting SPI and I2C master and slave modes.

### 3.1. Hardware Description of the RTU

#### 3.1.1. Acquisition and Processing Module (APM)

**Figure 2.**
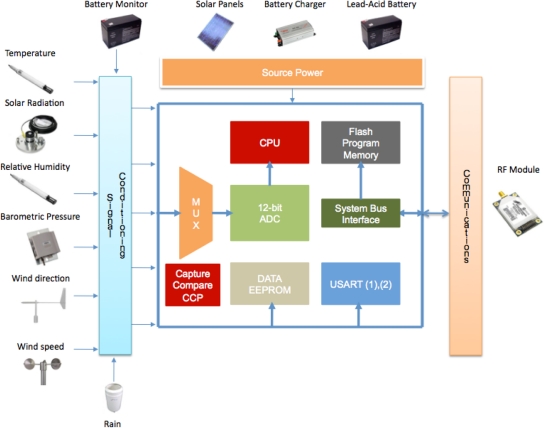
Block diagram of the main modules of the Remote Terminal Unit.

This module includes six analog channels (with possible expansion up to 16), which are used to receive signals from the climatologic sensors such as: AT, RH, SR, BP, WD and WS. It also monitors the state of the rechargeable battery in order to prevent a power failure. The RTU also processes two digital channels, which consist of signals delivered by the WS and rain sensor. The analog channels are designed to receive input voltages from 0 to 5 V (solar radiation sensor), 0 to 2.5 V (Pressure Sensor and voltage of the battery) and from 0 to 1 V (Temperature, Pressure, and Relative Humidity sensors). These voltages are converted into a 12-bits digital value by the internal ADC of the microcontroller and saved in the EEPROM data memory. Digital channels are related to pulse counting or frequency measuring. The rain sensor catches rainfall in a 200 mm collection funnel. When 25.4 mm of rainfall are collected, the tipping bucket assembly tips and activates a reed switch. As a consequence, the switch closure generates a pulse, which is recorded by the MCU and added in a 32-bits counter. Finally, when the bucket tips, the water drains out the screened base of the gage. On the other hand, wind speed is measured with a three-cup anemometer. Rotation of the cup wheel opens and closes a reed switch at a rate proportional to wind speed. The maximum frequency that the MCU can measure is 74.76 Hz or, in terms of speed, the range varies from 0 to 49 ms^−1^. In Figure 2 a block diagram of the main components of the APM are depicted as well as the source power and communication modules, which will be explained in the next section.

#### 3.1.2. Power Source Module

The power source is constituted of a single 20-W solar panel, an intelligent battery charger and a 12 V, 7 A lead-acid battery. The solar panel is the photovoltaic power source for the battery charger. Its characteristics assume 1 kW m^−2^ illumination and 25 °C solar panel temperature. It provides 20 W maximum peak power and 1.19 A of peak current, or enough to supply the acquisition and communications modules. The intelligent battery charger is based on the UC3906 integrated circuit. It monitors and controls both the output voltage and current of the charger through three separate charge states; a high current bulk-charge state, a controlled over-charge and a precision float-charge or standby state. The charging process, in other words, begins when the output current of the charger is limited to a low-level until the battery reaches a specified voltage (preventing a high current charging if a battery cell is shorted) then, the charger enters in a high rate bulk-charge state. In this state, the charger supplies a peak current until the battery voltage reaches 95% of its maximum capacity. Following, the charger changes to the over-charge state in order to charge completely the battery, in this state the charging current will be reduced to the minimum as the voltage approaches to its maximum capacity, and then, the charger will change to a floating or standby state. As the load (constituted of the acquisition and communication modules) is connected, if the battery drops 10% below the float level, the charger will reset itself and begin again the charging process.

#### 3.1.3. Communications Module

For wireless communication purposes between the RTUs and BTUs, RF Xtend modules from Digi were used. These modules allow an interface to a host device through TTL levels by using a USART or through an RS-232 interface. With these modules, a mesh network topology was implemented; this allows a message to be addressed to different nodes within the network until it reaches its final destination. In case of connection losses due mainly to weather conditions, critical data might still reach its destination due to programmed capacities in the RF modules which are listed below:

- *Self-healing:* Any node can join or exit the network at any time without this causes a failure in the whole network.
- *P2P architecture*: No hierarchy is recognized between RF modules.
- *Acknowledgements delivery*: Remote nodes will answer when a successful communication has been established increasing reliability in transmissions.

When a transmission starts, the following occurs:

1. A 10 bits preamble is sent (with the configured number of retransmissions).
2. Data are sent, beginning with the header (destination address type MAC address) and ending with a 16-bit CRC.
3. Wait for an acknowledgement in the MAC layer for 1.8 ms.
4. If the ACK is received, the transmission has been accomplished successfully.
5. Otherwise a timer is initialized and when it overflows a retry will be attempted.

**Figure 3.**
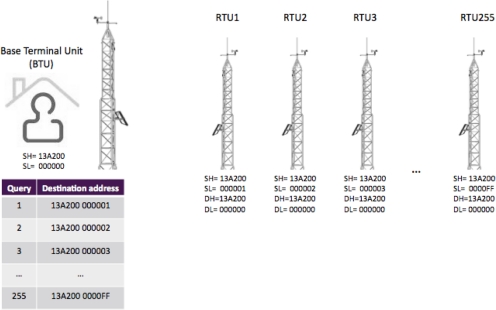
Example of destination and source parameters in wireless communication between remote and base units.

All the RTUs need to transmit data to the corresponding Base Terminal Units (BTUs). This is, unique parameters related to the serial number of each terminal unit are considered: (a) Destination address constituted by high and low part (DH and DL respectively) and (b) Source address constituted by high and low part (SH and SL respectively). Making an analogy to a telephone system, the destination number (DH-DL) that the RTUs have to “dial” is the BTU’s unique serial number (SH-SL). On the other hand, to communicate the BTU with each one of the RTUs, it will be necessary to “dial” the unique serial number (SH-SL) of each one of the RTUs. This address relationship is shown in Figure 3.

### 3.2. Embedded Software in the RTUs

An embedded software is typically based on a microcontroller or digital signal processor (DSP) which has to perform one or more dedicated functions (commonly in real time). The embedded software which runs in the MCU PIC18F8723 is programmed in C language and using the compiler from Custom Computer Services (CCS). The most important routine is described below.

#### 

##### Master Routine

The RTUs’ master routine is predominantly controlled by interruptions, this means that the tasks that the system performs are initialized by different types of events. Among the used interruptions can be found: CCP Interruption (Capture-Compare interruption related to the measurement of the frequency of the wind speed sensor), External Interruption (related to measure the amount of rain fall) and the USART interruption (activated when climatologic data is requested to the RTUs).

**Figure 4.**
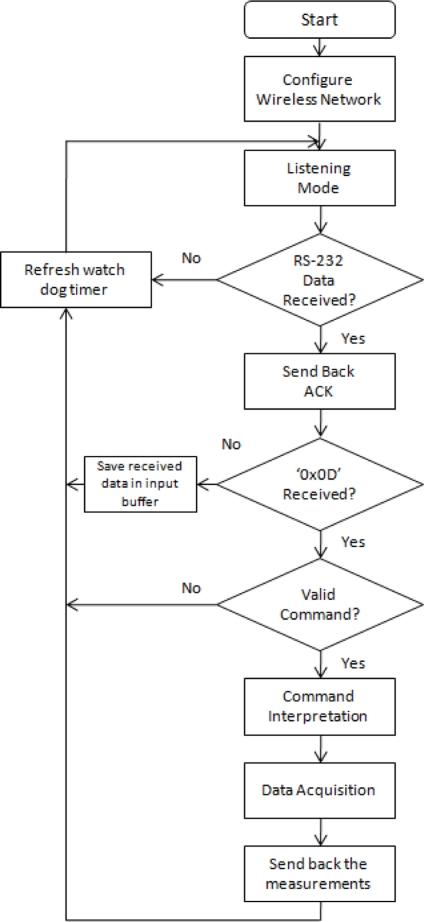
Flow diagram of the master routine.

The communication model that has been used is a master-slave type; this is, the slave is always in “listening” mode and only transmits when it is requested. The master is the central server that generates a request to the BTU, which identifies the destination of the query and requests the remote data from the RTU (which remains in “listening” mode). This model reduces significantly the traffic inside the wireless network because the master controls all the transmissions within the network. The flow diagram of the main routine inside the RTUs is illustrated in Figure 4.

The main routine configures the wireless network at first. This stage consists on setting up: Destination address (DH-DL), Network retries (NR), Power level (PL), Hopping Channel (HP) and Network hops (NH). Then, the MCU enters to listening mode; this is, waits for data on the Tx pin of the USART module. If no data is collected, the main routine will refresh the watchdog timer to keep the MCU in normal mode operation, otherwise an acknowledgement of data received is sent to the base. When the character “Enter” (0x0D hexadecimal) is received the program will consequently validate the characters previously stored in the input buffer and if validation occurs, a command interpretation will follow according to the following Table 1.

After the command interpretation, data acquisition is achieved by requesting the values to the corresponding modules (in terms of voltage, frequency or counts) and then converted to physical units in a two-digits format (XX.XX units). Finally weather measurements are sent back to the base and the watchdog refreshed.

## 4. Remote Base Unit

This remote unit controls when and who can transmit within the wireless network. It is based on the high performance microcontroller PIC18F6722 and is constituted of three main modules: power source module, RF communications and HTTP Embedded server. In distinction to the RTUs, there will be only one BTU for each wireless sensor network. In the next sections BTU’s hardware and software is described.

### 4.1. Hardware Description of the Remote Base Unit

**Figure 5.**
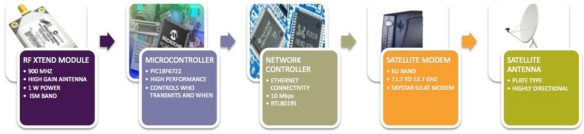
Main components of the Remote Base Unit.

The main function of the BTU consists of decoding the data acquisition requests originating in the central server and perform the corresponding queries to each one of the RTUs within the wireless network. Thus, the RBU acts as an entry/exit point to the network, in other words, joins together two networks that use different base protocols, in one side a WSN (the one constituted by the RTUs) transmitting in a 900 MHz frequency using the band ISM (intended for Industrial, Scientific and Medical applications) and on the other side the a WAN interconnected by satellite networks. In Figure 5, the main blocks of a Remote Base Unit are illustrated.

**Figure 6.**
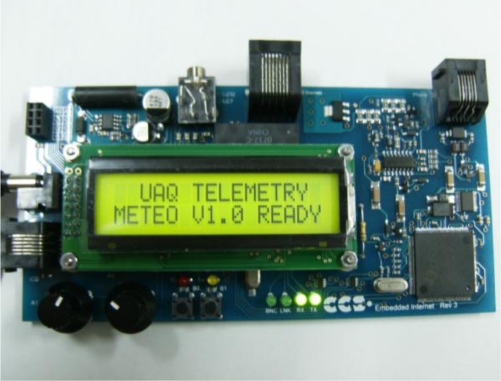
Embedded HTTP Server of base terminal unit.

The satellite module is constituted by a Skystar 360E modem, which receives frequencies between the ranges from 11.7 to 12.7 GHz, decode and send them through a 10 Mbps Ethernet link. By using a category 5 UTP cable and RJ45 connectors, these signals are transmitted to an embedded RTL8019S network controller of the HTTP server which decodes the Manchester line code used by Ethernet and send them by using a SPI bus to the PIC18F6722 microcontroller (MCU). The MCU then, examines these queries and sends the corresponding commands to the RF Xtend module via RS232. Once the RTUs acquire data, they send the information back to the base which will retransmit it to the CS. In Figure 6, a photograph of the Embedded HTTP server is shown.

#### 4.1.1. Interface with the RF Module

**Figure 7.**
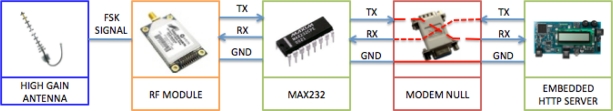
Connections between the Embedded HTTP Server and the RF Module.

The HTTP server includes a RS232 port, which allows communication between the PIC18F6722 and the RF module. Necessary hardware to connect both devices includes an integrated circuit MAX232 (for converting RS232 to USART protocol) and a modem-null module (wired to interconnect transmission Tx with reception terminals Rx and viceverza). The connections’ diagram is shown in Figure 7.

#### 4.1.2. Satellite Network

In the proposed system, wireless long range monitoring was desired. To achieve this, it has been chosen Ku band transmissions over a satellite network.

##### Operating Principle

**Figure 8.**
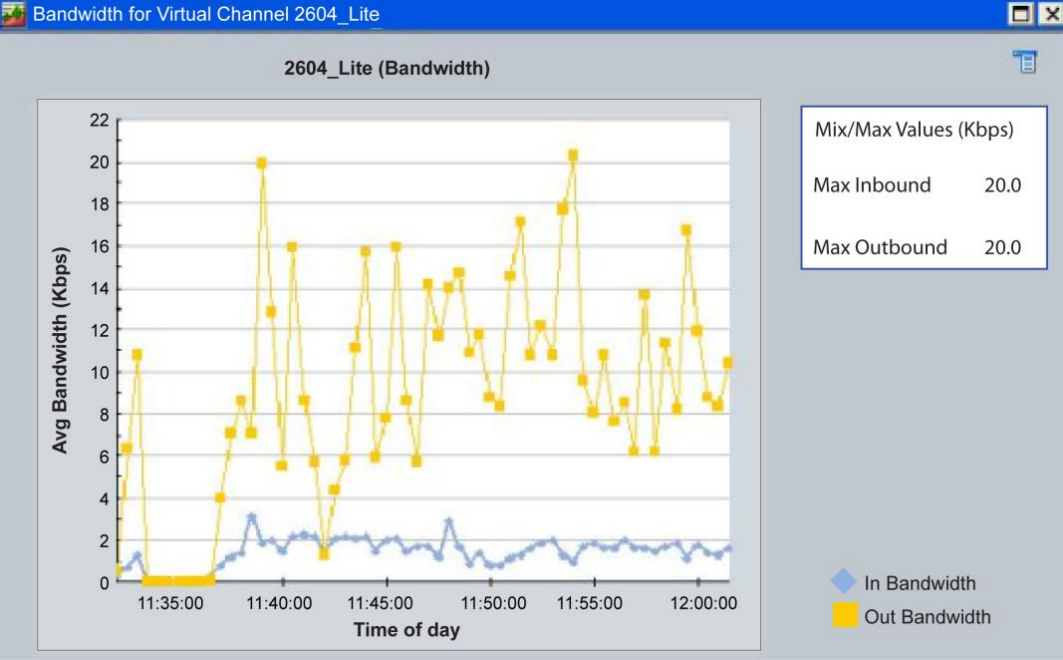
Bandwidth required in a satellite link for transmitting meteorological data.

Information is requested from the central server through a “get” command on an HTTP webpage, then, information will pass through a gateway to be interconnected to a public network. After that, a Very Small Aperture Terminal (VSAT) will send this information to a satellite KU band. This band has the advantage of using lower power transmitters but with the inconvenience of low rate data transmission. However, in order to test the inbound and outbound data rates, preliminary tests were performed having the system transmitting each ten minutes the collected data of climatic variables. This test is shown in Figure 8.

In Figure 8 it can be appreciated that the maximum inbound data rate (from the RTU to the satellite) is around 3.5 Kbps, while the maximum outbound data rate (from the satellite) to the Central Server is around 20 Kbps. With this test, it was proved that bandwidth used for the system was really low, in addition if this information is requested only every ten minutes, the amount of data transmitted is minimum, so that, a low-speed satellite link was rented from a private ISP.

#### 4.1.3. Source Power Module of the Base Terminal Unit

**Figure 9.**
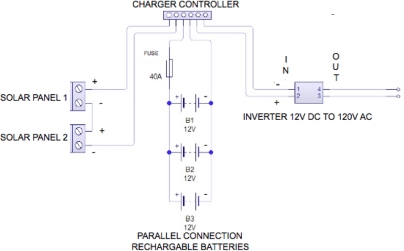
Connection diagram of the power source module for the base terminal units.

This module is really important for the long range telemetry systems in adverse geographic conditions because most of the times there is no electricity available to power measurement equipment and satellite links. Two 120-W solar panels connected in series generate the input voltage for a charge controller which has two main functions; first, to charge three 12-V deep cycle batteries until its optimum level and second, supply 12 V to the DC-AC inverter which powers the satellite modem. The connection diagram is shown in Figure 9.

### 4.2. Software of the Base Terminal Unit

**Figure 10.**
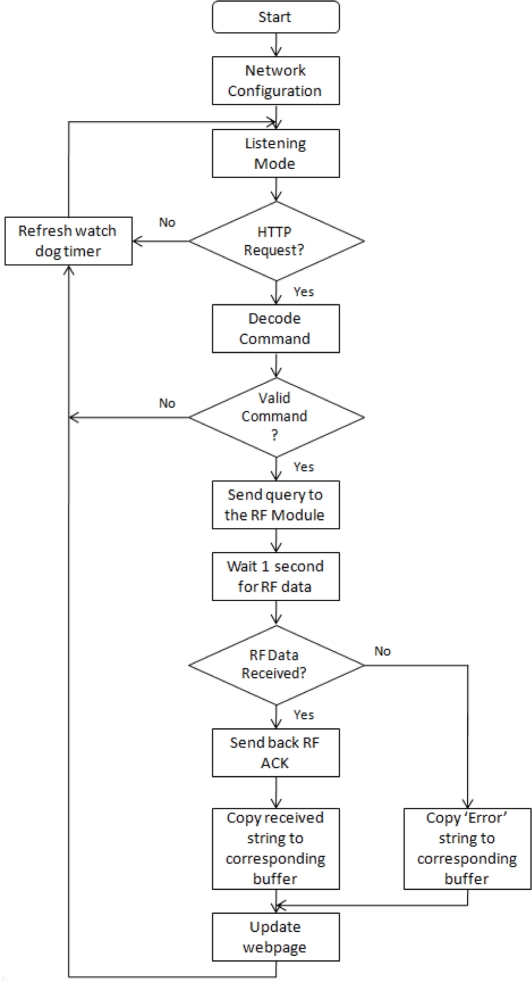
Flow diagram for the main routine in the remote base unit.

The same as for the RTUs, the BTU’s software is programmed in C language and the C compiler from CCS is used. The embedded software performs data acquisition and communication with the RF module and with the HTTP server. The main routine configures the network parameters: IP Address, Subnet Mask and Gateway in order to establish communication from the HTTP server to the satellite modem. Then, the program waits for a query from the Central Server in form of an http request to retrieve an HTML page. Within the web page, the special formatting character “%” is found. When an http task is serving a webpage and finds a “%” it calls a function with the ID variable set to the formatting character. For example in the html code of the webpage programmed in the PIC18F6722 it can be found: *<h4>Temperature: <!-- -->%7 ºC<br>*, where the “ID” of the variable requested is the number “7” which follows the special character. If the ID received is valid, the main routine will send a query through RS-232 port to the RF module and then, will wait one second for the data to arrive. If that happens it will send back an acknowledgement to the RTU and copy the received characters to a corresponding buffer, otherwise the string that will be copied to the buffer will be “Error”. After the acquisition, the webpage will be updated with the received data and the program will wait for another http request. This is illustrated in Figure 10.

## 5. Central Server (CS)

The CS has the main task to manage web consulting/data downloads request generated by the end users, to maintain updated data and generate historical records. When a final user asks for data in the telemetry system through an Internet browser, a request is generated to the CS, then it shows the stored data and updates it each ten minutes.

### 

#### 

##### Remote Data Acquisition

Remote Data Acquisition involves two main parts: the client (represented by the BTU’s HTTP embedded server) and the CS, which manages the meteorological data from the remote stations and provides information to the database for further analysis and to be displayed in real-time on the web portal.

In the central server a program in PHP language which requests the meteorological data to the base terminal units every ten minutes is stored. To achieve this, an infinite-loop routine monitors every second the time of the CS by using the conditioning sentence that is expressed as follows:

(min % delay==0)&(sec==0)

In the above line of code, “min” represents the minutes value from the clock of the system, the variable “delay” is previously set by the user and defines the sampling period in minutes (10 by default) and “sec” is the variable which contains the seconds value from the clock of the system. If this condition is not true, the system continues updating the variables “min” and “sec”. Otherwise if the condition in the above flow diagram is true, the program executes the acquisition routine, which performs previous corroborations before attempting to connect to the RTUs.

**Figure 11.**
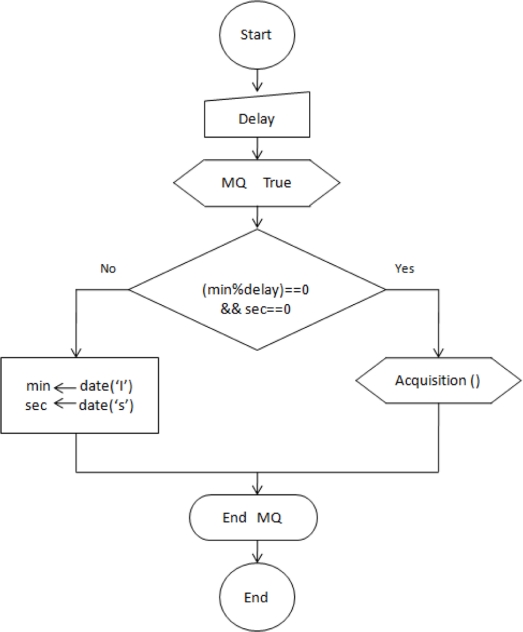
Flow diagram for the data transmission request in the central server.

1. Consults the state of the flag “enabled” to know if the remote station must be consulted (for example, remote stations can be disabled in case of maintenance). On the other hand, in case that the state of the flag is false, the label “NEBLE” will be added in all the fields of the corresponding database in the central server.
2. If the state of the flag is true, then the status of the Internet connection is checked by using a “ping” command to the IP address 66.102.7.99 (Google server). In other words with a ping command expects an eco response and if it is bigger than zero, the main program will try to establish connection with the RTU. Otherwise in all the fields of the corresponding RTU will be added the label “NNET”. This means that the Internet connection has been lost.
3. An information request is sent to the BTU by using the “get” method, which tracks keywords or symbols along the embedded webpage in the BTU. For example to read the relative humidity in a base terminal unit, the “get” command “sweeps” the webpage content and looks for the words “Relative” plus “blank space” plus “Humidity”. After that, the number that follows the keywords is read. The flow diagram of the requests generated by the CS is shown in Figure 11.

## 6. System Testing and Results

### 6.1. Preliminary Tests

**Figure 12.**
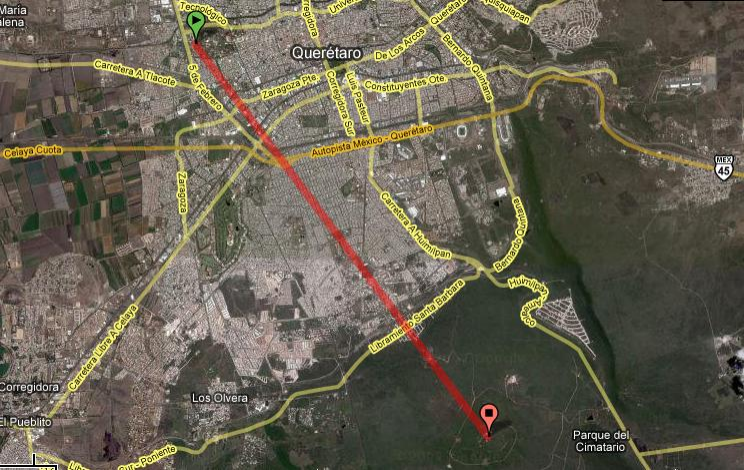
Preliminary field test in the City of Querétaro.

Several tests have been performed at different stages of the development of the system. In relation to the radiofrequency communication system, the first test consisted on a loop-back type communication between a BTU and a RTU in line of sight. In this test, the BTU sends data packets and the RTU has to return exactly the same values. These preliminary tests confirmed a successful communication in a distance of 8.77 Km in line of sight (LOS) from the engineering school to the mountain “Cerro del Cimatario” near the city of Querétaro, as illustrated in Figure 12.

**Figure 13.**
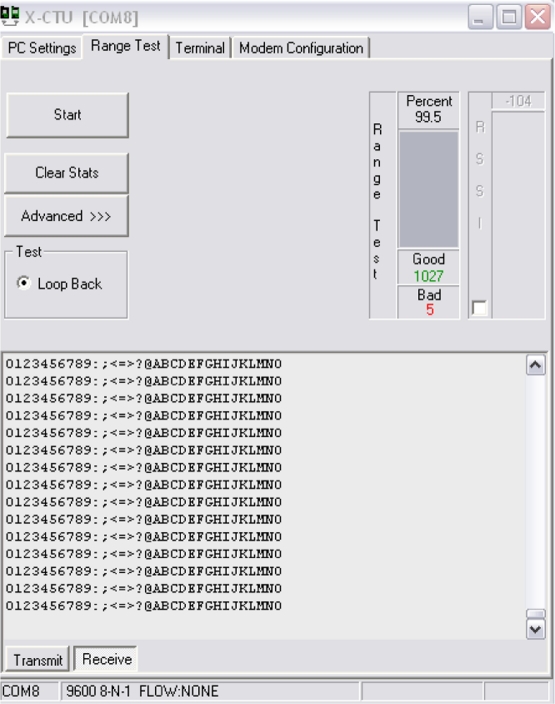
Range test with the X-CTU software.

The LOS analysis was performed by using the X-CTU software from Digi. Data packets sent consisted of a sequence of alphanumerical characters, as shown in Figure 13, and transmitted at a rate of 9,600 bps. The results of the range test showed a 99.5% of reliability, which in other words means that for 1,000 transmitted packets, only five were received incorrectly.

With the obtained results, the next step consisted of performing field communication tests in 11 different points at “Los Altos de Chiapas” (Table 2), a mountainous zone of the southeast of Mexico where the system is currently implemented. LOS and range tests where achieved and the relation between packets sent/received correctly were calculated. In conditions of LOS, the communication system had a maximum error of about 0.25%. For example in “Agua Prieta”, there was an error of only 0.24%, that is 1,249 packets were transmitted and only three were received incorrectly.

**Figure 14.**
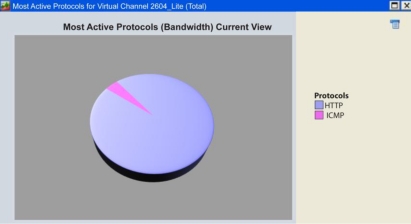
Most active protocols during data transmission when using satellite link communication

At this point, only tests of the transmission of the wireless RTU’s network in a loop-back mode have been shown, however it is important to evaluate the possible errors generated from a request generated by the final user through a web browser, the reception of the request by the CS, the correct data transmission through the satellite network and then the decoding of the query before this is sent to the wireless RTU’s network. For this purpose, a monitoring of the most used protocols in a loop-back data transfer was performed. In Figure 14, two protocols are depicted: the Hypertext Transfer Protocol intended for client-server transmissions where the client submits http request (case of the final user requesting weather information) and the server responds with messages to it (case of the HTTP server sending back the acquired data by the RTUs), and the other, the Internet Control Message Protocol (ICMP).

The ICMP is the sub-protocol of control and error notification of the Internet Protocol (IP) used to send error messages indicating for example, that a predefined service is not available or a router can’t be addressed. In Figure 14, it can be appreciated that the HTTP utilizes about a 97% of the available bandwidth and the ICMP protocol only the remaining 3%. This result indicates a high reliability in the satellite link and in the transmissions managed by the HTTP embedded server.

### 6.2. Web Portal for the Final User

A Web portal which contains a map of the region where the system is currently implemented, web pages to consult the climatic variables in each one of the RTUs, a downloads zone and general information of the system is stored in the CS located at the Engineering School of the Universidad Autónoma de Querétaro.

**Figure 15.**
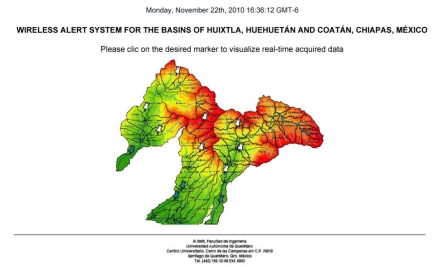
Main screen of the web portal which shows the location of the RTUs in the geographic region “Los Altos de Chiapas”, México.

The main screen of the web portal is shown in Figure 15, where the RTU’s location map can be appreciated in the center. The specific location of each one of the RTUs is divided between three wireless stations networks. One is located in the upper left of the map and constituted by the EMA Cerro de las Cruces, EMA Finca la Victoria and EMA Agua Prieta. The second can be found at the center and is formed by the EMA Chanjul and the EMA Pinabeto. Finally a third wireless stations network constituted by the EMA Buena Vista, EMA Piedra Parada and the EMA Vega de los Gatos.

**Figure 16.**
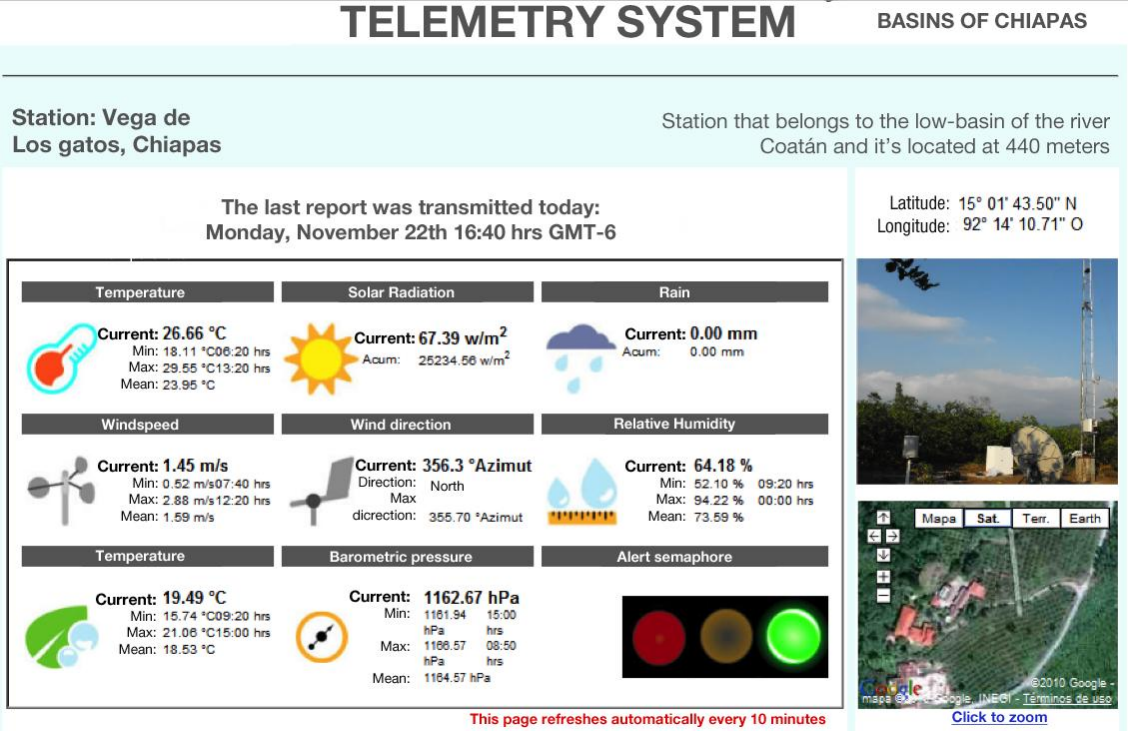
Numerical interface oriented to the final user.

When the final user clicks on a specific RTU located in the map, the user is redirected to a webpage (Figure 16) where numerical weather information is shown, and also the charge level of the battery is shown in order to schedule maintenance or to prevent a power failure. On the left side a photograph of the RTU *in situ* and a Google’s Earth application to visualize satellite pictures of the site can be appreciated.

**Figure 17.**
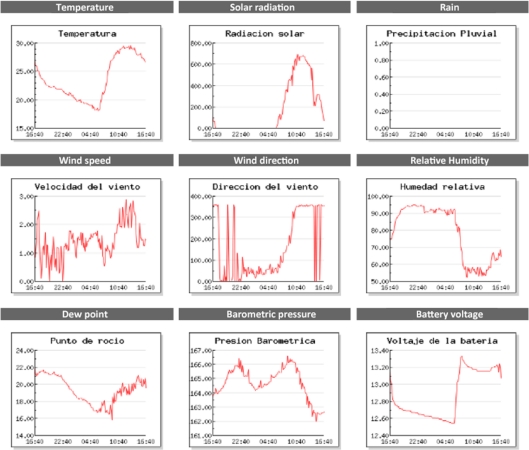
Graphical representation of the acquired data.

On the same RTU’s webpage, a graphical interface is presented. In this, records of the collected data in the last 24 h are shown in a scrolling graph. This is, the last measurement acquired is graphed on the extreme right side and with the arrival of new data it moves to the left side, thus, after 24 h this data will not be shown any more. In Figure 17 an example of the graphical representation of the acquired data is shown.

**Figure 18.**
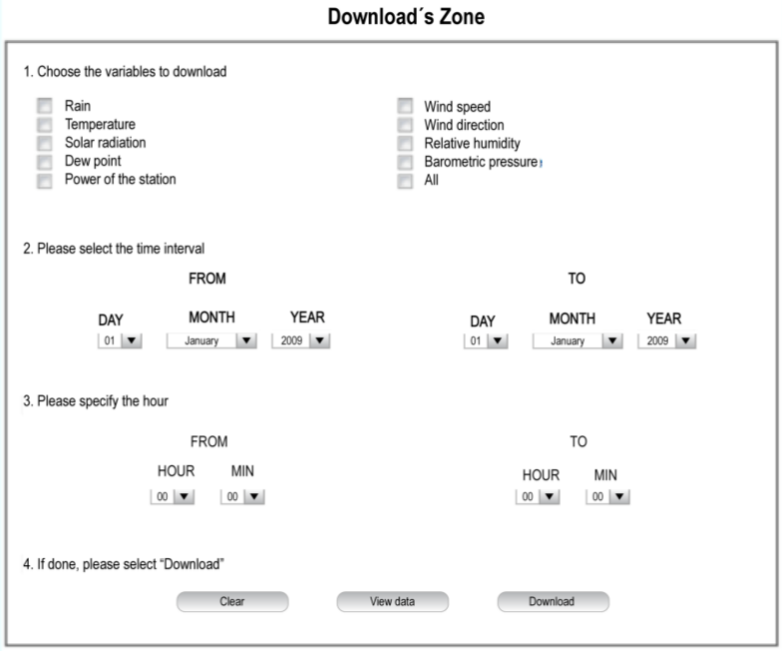
Downloads zone oriented to the final user.

In addition to the numerical and graphical representation of the measurements, it is possible to download acquired data from a download zone shown in Figure 18. There are only four steps that the final user needs to follow to download data from the CS: choose the variables to be downloaded in a text file, select the date interval, decide on the specific time interval and click on “download data”.

**Figure 19.**
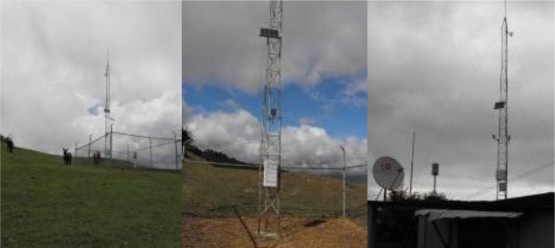
Photographs of the installed RTUs.

Lastly, in Figure 19 photographs of the RTUs (left and center) as well as the BTU (right) are shown.

## 7. Performance of the WLAN

**Figure 20.**
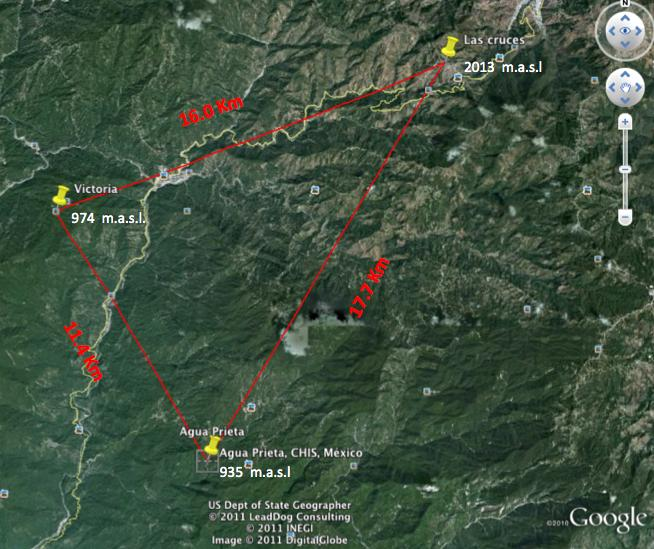
Distances measured between RTUs and BTU in Huixtla’s basin.

Because of the geography of the zone and as a result of a Line of Sight (LOS) analysis, the WLAN was divided into three different zones, “Huixtla”, “Coatán” and “Huehuetán”. To exemplify the performance of the network, the distribution of the Huixtla zone is shown in Figure 20. The distances from point to point correspond: 17.7 Km from Agua Prieta to Las Cruces; 11.4 Km from Agua Prieta to Victoria and 16 Km from Victoria to Las Cruces. At this point is important to say that those distances are reached using fiberglass, omni-directional high-gain 15″ base station antennas with 2.1 dBi gain, which reach up to 64 Km.

The main contribution of the proposed architecture relies on the capacity to deliver reliable information even under adverse weather conditions such as rainstorms. This can be explained as follows. Based on Figure 20, let’s suppose that a rainstorm approaches the path between Agua Prieta and Las Cruces interfering with communication between both units. When this is detected, the wireless network will automatically find a different route to reach the base unit. This is, if after the maximum number of retries (three) the BTU (Agua Prieta) has not received any acknowledgement from the RTU (Las Cruces), the BTU will automatically request another RTU (Victoria) to establish a link to Las Cruces to gather the information. When this occurs the system will change the following parameters: (a) Data receive timeout will be changed from 2,000 ms to 4,000 ms (it was measured that each hop takes about 1,800 ms), due to the hops number will be doubled; (b) Destination address in the isolated RTU (Las cruces) will change to the available RTU which will be used as a bridge (Victoria in this case); and (c) The RTU that establishes a communication link with the isolated RTU will be reconfigured as a repeater, this is, will “listen” to all the stations and retransmit the information to the base. The same will happen with interferences in all the routes between BTUs and RTUs in the three different basins.

## 8. Conclusions

A long-range remote monitoring system constituted by three parts: Remote Terminal Units (BTUs), Base Terminal Units (BTUs) and a Central Server (CS) has been developed. Field tests have shown a high reliability (97%) of the satellite network data transmission and in the LOS analysis in the 900 MHz wireless networks it was 99.8%. The WSN currently implemented in the region “Los Altos de Chiapas” reduces the need of satellite links for each RTU due to the use of 900 MHz RF transmitters in the ISM band intended for Industrial, Scientific and Medical applications and as a consequence the implementation cost is minimized. Although the monitoring system is currently focused on the measurement and transmission of climatologic variables, it is designed following the principle of modularity, so it is possible to change the network sensors and apply it in multiple applications in the industrial, scientific and medical sectors and generally speaking in any application where the remote monitoring represents some danger, or could be inaccessible or economically non-viable for the user.

## Figures and Tables

**Table 1.** List of commands decoded by the Remote Terminal Units.

**Command**	**Description**	**Module**
T,0 × 0d,0	Temperature	Analogical 0
H,0 × 0d,0	Relative Humidity	Analogical 1
P,0 × 0d,0	Barometric pressure	Analogical 2
S,0 × 0d,0	Wind speed	Module CCP
R,0 × 0d,0	Solar Radiation	Analogical 4
L,0 × 0d,0	Rain	External Interruption
D,0 × 0d,0	Wind direction	Analogical 5
Z,0 × 0d,0	Restarts remotely the MCU	N.A.
C,0 × 0d,0	Dew point	N.A.
V,0 × 0d,0	Battery voltage	Analogical 10
A,0 × 0d,0	Set up the real time clock	Module I^2^C

**Table 2.** Field measurement results in the selected RTU installation points.

**Test point ID**	**Test site name**	**GPS coordinates**	**Reference test point**	**Line of sight Yes/No**	**RSSI %**	**Good packets**	**Bad packets**
1	Las Cruces	577062 E	2	YES	99.8	1,251	2
		1695731 N					
2	Las Cruces	577062 E	1	YES	100	2,000	0
		1695731 N					
3	Nueva	563612 E	1	YES	99.8	1,251	2
	Victoria	1690648 N					
4	Agua Prieta	568104 E	1	NO	0	0	1,250
		1682104 N					
5	Niquivil	582519 E	1	NO	0	0	1,250
		1684780N					
6	Nueva	563612 E	6	YES	100	2,000	0
	Victoria	1690648 N					
7	Agua Prieta	568104 E	6	YES	99.8	1,249	3
		1682104 N					
8	Niquivil	582519 E	6	NO	0	0	1,250
		1684780N					
9	El	584262 E	9	YES	100	2,000	0
	Chaparrón	1672452 N					
10	Niquivil	582519 E	9	NO	0	0	1,250
		1684780N					
11	Unión Roja	584125 E	9	NO	0	0	1,250
		1663839 N					
